# Short-Term Changes in Mental, Physical, and Social Factors After Metabolic Bariatric Surgery in Adolescents: A Nationwide Prospective Cohort Study

**DOI:** 10.3389/fnut.2022.878202

**Published:** 2022-05-12

**Authors:** Ariela Goldenshluger, Tamar Maor, Renana Via-Kagan, Orly Zelekha, Yftach Gepner

**Affiliations:** ^1^Department of Epidemiology and Preventive Medicine, Sackler Faculty of Medicine, School of Public Health, Sylvan Adams Sports Institute, Tel-Aviv University, Tel-Aviv, Israel; ^2^Israel Center for Disease Control, Ministry of Health, Jerusalem, Israel

**Keywords:** bariatric surgery, physical status, adolescents, obesity, mental health, social rejection

## Abstract

**Background:**

Metabolic bariatric surgery (MBS) is an effective treatment for adolescents with severe obesity. However, changes in mental, physical, and social factors, as well as their association with the extent of excess weight loss (%EWL) after MBS, remain controversial.

**Methods:**

We followed 97 adolescents (64% females, aged 17 ± 0.9 years, BMI 46.1 ± 5.9 kg/m^2^) before and 9 months following MBS in a multi-center, prospective cohort study. Changes in mental, physical, and social factors were assessed by self-reported questionnaires, and associations with %EWL were evaluated after adjustment for potential confounders.

**Results:**

The body mass index (BMI) decreased by 30%, and all physical parameters significantly improved (*p* ≤ 0.001). Energy level increased by 24%, mood level by 14%, and mental health by 9.5% (*p* ≤ 0.002). Social parameters were also improved, with a significant decrease in social rejection (*p* = 0.02), and an increase in participation in after-school social activities (*p* = 0.008). Mental health improvement was associated with baseline social rejection (r = 0.514, *p* < 0.001). The improvement in all factors was not related to the extent of %EWL.

**Conclusion:**

Metabolic bariatric surgery MBS in adolescents led to a meaningful decrease in BMI and to an improvement in short-term physical, mental, and social factors that were not related to %EWL. Patients experiencing social rejection may improve their mental health following BS.

## Introduction

The prevalence of severe obesity [BMI of ≥35 or >120% of the 95th percentile] has increased in the last decades among adults and adolescents (1–3) and remains a worldwide public health concern (4). Although lifestyle modification is a beneficial strategy to decrease some extent of body weight (5–7), metabolic bariatric surgery (MBS) has been found as a beneficial intervention for severe obesity and its related comorbidities in the long term for adolescents (7–9). A systematic review of 49 studies (*n* = 3,007) of adolescents undergoing MBS and long-term, multicenter, and prospective trials reported a 26–31% reduction in BMI, and significant remission of diabetes (86–100%), dyslipidemia (64–83%), and hypertension (68–92%) (7, 10–12). Despite the evidence for metabolic health benefits from MBS among adolescents, these candidates are a high-risk group. Since, adolescents with obesity are more likely to endorse lower self-esteem, depression, reduced quality of life, and anhedonia (13, 14), and even it has been shown that mental factors could affect the chances to perform MBS and complete the preoperative program (14). Nationally, it is recommended an optimal multidisciplinary follow-up including visits to the bariatric surgeon, dietitian, and pediatrician for all patients, and a mental health professional follow-up according to personal need (15). Following surgery, the quality of life (QoL) in adolescents has been associated with long-term weight loss maintenance (16). Previous studies have reported an improvement in QoL in adolescents from 1 to 5 years after MBS (10, 11, 17, 18). However, 5 years following MBS, the improvement in QoL was mainly physical, not mental (19). The association between changes in mental, mental, and physical factors and weight loss and changes after MBS in adolescents remains unclear. Therefore, the objectives of this study were to evaluate the mental, physical, and social factor changes from baseline and to assess the association between those changes and EWL%, using a nationwide prospective cohort of adolescents after MBS.

## Methods

This prospective cohort study was conducted among adolescents who underwent MBS at 21 different medical institutions in Israel, between October 2017 and December 2019, using data from the Israeli National Registry of Adolescents Bariatric Surgery, a mandatory registry created by the Ministry of Health, in which all bariatric surgeries performed in adolescents in Israel, are registered. The registry includes data collected at a baseline visit in the bariatric Clinic before the surgery and the follow-up, performed by self-reported telephonic questionnaires. All patients and their families were provided informed consent for the study. The study was approved by the Institutional Review Board number of the Tel HaShomer Medical Center (6199-19-SMC) and by the Tel Aviv University's Helsinki committee (0001814-1).

### Mental, Physical, and Social Factors

All study participants were requested to complete a baseline questionnaire before surgery regarding physical and social functioning and mental health factors. The questionnaire (Supplementary Material 1) is mandatory at baseline as part of the National Bariatric Registry, which gathers information about every adolescent undergoing bariatric surgery in the country. National guidelines for MBS in patients under the age of 18 years, are part of the Ministry of Health's medical director notice 27/2017 (August 2017) (15) and encourage follow-up of adolescents undergoing bariatric surgeries in Israel. The questionnaire included sets of questions on health status (e.g., obesity-related co-morbidities, chronic pain related to weight, smoking habits, and substance use), physical (energy level, and hygiene-related difficulties), mental (sadness, hopeless feeling, low self-esteem, and history of self-hurt attempt) and social factors (social rejection, after-school activities participation), as well as sets of questions on dietary patterns (vomiting, overeating episodes, loss of control eating, and eating at night, dietitian visits). Parents and adolescents (in the presence of a parent and with given permission) completed the questionnaire during the preoperative visit to the bariatric clinic. Preoperative presence of comorbidities was contributed by a physician at the bariatric medical center and based on medical records. The follow-up telephonic questionnaire was completed by Israeli National Registry of Adolescents Bariatric Surgery research assistants and the research team interviewers, at a mean of 8.8 ± 3.4 months post-surgery.

Questions regarding “Energy level” and “Mood level” have five-point Likert scale answers and were converted to a 0–100 scale. Dichotomous questions (yes/no) regarding physical state (e.g., snoring, pain affected by weight, physical activity habits), social factors (after-school social activities and social rejection), and mental health factors (sadness, low self-esteem, hopelessness, suicidal thoughts, and self-hurt attempts history) were performed.

The mental health questions were grouped as a mental health score with a higher score on a 0–100 scale meaning a better status (Cronbach's Alpha of 0.67).

### %EWL Calculations

The %EWL was calculated by using the formula:


$$\begin{array}{l} \%EWL=\frac{\left[\left(initial\;weight\right)-\left(post\;operativeweight\right)\right]}{\left[\left(initial\;weight\right)-\left(ideal\;body\;weight\right)\right]}\times 100 \end{array}$$

The ideal body weight was defined by the weight corresponding to a BMI of 25 kg/m^2^, according to the American Society for Metabolic and Bariatric Surgery outcome reporting standards (20). The initial weight and height were taken from the measurement by the medical center during the preoperative visit, and the post-operative weight was self-reported at the interview.

### Statistical Analysis

Baseline characteristics of those who agreed to be interviewed after surgery and those who refused were compared by a *t*-test or Chi-square test for continuous and categorical variables, respectively. Changes in mental, physical, and social factors were examined by a paired *t*-test for numeric variables and by a McNemar test for dichotomous variables. The association between factor changes and %EWL was assessed using the Pearson correlation for numerical variables. In addition, we divided the participants into below and above median %EWL [(%EWL <67% (*n* = 48), and %EWL ≥ 67% (*n* = 46)] and compared the change in mental, physical, and social factors between weight loss categories by *t*-test for numerical variables and by a Chi-squared test for dichotomous variables. A univariate linear regression model was used to evaluate the association between dependent variables of mental, physical, and social factors changes (for numerical parameters) and potential independent variables: age, gender, baseline BMI, ethnic group, dietitian meetings number, and number of comorbidities. A multivariate linear regression model assessed associations between significant variables in the univariate analysis and was adjusted for possible confounders: age, gender, and baseline BMI or gender, baseline BMI, and time from surgery. Additionally, a sensitivity analysis of the self-reported weight, before surgery compared to documented weight at hospitalization, was performed. All statistical analyses were two-sided with a significance of *p* < 0.05.

## Results

In total, 107 out of 123 adolescents who underwent MBS from 10/2017-to 12/2019 were contacted and 16 refused to participate. We excluded five participants, who were less or beyond the follow-up time frame, and five participants who underwent additional surgery after the MBS. In total, 97 adolescents were interviewed at 8.8 ± 3.4 months post-surgery. Baseline characteristics are summarized in Table 1 by gender (62 females and 35 males, aged 17 ± 0.9 years, with a BMI of 46.1 ± 5.9). Sleeve gastrectomy was the most common surgical procedure (86.6%). The dietary patterns report showed a sense of loss of control while eating (*n* = 38, 40.4%), night eating (*n* = 48, 51.1%), excess eating (*n* = 47, 50%), and vomiting (*n* = 3, 3.2%). Females had lower systolic blood pressure, lower rates of hypertension diagnosis, and higher rates of Attention-Deficit Hyperactivity Disorder (ADHD) (*p* = 0.003, *p* < 0.001, and *p* = 0.004, respectively). Except for gender, there were no other significant differences in baseline characteristics between participants and those who refused to be interviewed (Supplementary Material 2).

**Table 1:** Baseline characteristics of the participants before metabolic bariatric surgery.

		**Male**	**Female**	**Total**	***P***
		**(*n* = 35)**	**(*n* = 62)**	**(*n* = 97)**	
**Demographic characteristics**
Age, years		17.1 ± 0.9	17.0 ± 0.9	17.0 ± 0.9	0.58
Ethnic group, *n* (%)	Jewish	31 (88.6%)	51 (83.6%)*^*a*^*	82 (85.4%)*^*a*^*	0.51
	Arabic	4 (11.4%)	10 (16.4%)*^*a*^*	14 (14.6%)*^*a*^*	
**Clinical characteristics**
Surgery procedure, *n* (%)	Sleeve gastrectomy	31 (88.6%)	53 (85.5%)	84 (86.6%)	0.76*
	Roux-en Y gastric bypass	0	2 (3.2%)	2 (2.1%)	
	Single anastomosis gastric bypass	3 (8.6%)	7 (11.3%)	10 (10.3%)	
	Gastric band	1 (2.9%)	0	1 (1%)	
BMI, kg/m^2^		45.4 ± 5.5	46.5 ± 6.1	46.1 ± 5.9	0.39
Comorbidities:					
Diabetes, *n* (%)		2 (5.7%)	8 (12.9%)	10 (10.3%)	0.32**
Hypertension, *n* (%)		12 (34.3%)	3 (4.8%)	15 (15.5%)	<0.001
Dyslipidemia, *n* (%)		12 (34.3%)	17 (27.4%)	29 (29.9%)	0.48
Fatty liver, *n* (%)		26 (76.5%)*^*a*^*	39 (62.9%)	65 (67.7%)*^*a*^*	0.17
Obstructive sleep apnea, *n* (%)		7 (20.0%)	18 (29.0%)	25 (25.8%)	0.33
Urinary incontinence, *n* (%)		0*^*e*^*	3 (5.2%)*^*d*^*	3 (3.4%)*^*f*^*	-
Orthopedic *^¥^, n* (%)		8 (22.9%)	14 (22.6%)	22 (22.7%)	0.97
Reflux, *n* (%)		6 (17.1%)	4 (6.5%)	10 (10.3%)	-
Anxiety, *n* (%)		0*^*c*^*	4 (6.6%)*^*a*^*	4 (4.3%)*^*d*^*	–
Depression, *n* (%)		1 (3.0%)*^*b*^*	4 (6.6%)*^*a*^*	5 (5.3%)*^*c*^*	–
ADHD, *n* (%)		4 (12.1%)*^*b*^*	24 (40.7%)*^*c*^*	28 (30.4%)*^*e*^*	0.004
Smoking, *n* (%)		5 (14.7%)*^*a*^*	6 (10.0%)*^*b*^*	11 (11.7%)*^*c*^*	0.52**
SBP, mmHg		133 ± 16	123 ± 13*^*a*^*	127 ± 14*^*a*^*	0.003
DBP, mmHg		72.8 ± 10	72.7 ± 11*^*a*^*	72.8 ± 11*^*a*^*	0.96
Irregular periods, *n* (%)		–	21 (33.9%)*^*a*^*	–	–
PCOS, *n* (%)	-	–	7 (11.3%)	–

*Data in the table are presented as mean ± SD for numerical variables and n (%) for dichotomous variables. t- test was used to evaluate differences for numerical variables and Chi–square test for nominal variables. All statistical analyses were two sided with a significance of <0.05. Irregular periods and PCOS were examined only for females*.

*calculated as sleeve gastrectomy vs. other procedures by Fisher's exact test.

**calculated by Fisher's exact test, ^a^1 participant is missing, ^b^2 are missing, ^c^3 are missing, ^d^4 are missing, ^e^5 are missing, ^f^9 are missing.

*ADHD, attention deficit and hyperactivity disorders; BMI, body mass index; SBP, systolic blood pressure; DBP, diastolic blood pressure; kg, kilograms; m, meters; mmHg, millimeters of mercury; PCOS, polycystic ovary syndrome. ^¥^Orthopedic pain related to body weight*.

### Mental, Physical, and Social Factors and Weight Changes After Surgery

Mean weight loss was 38.6 ± 11.2 kg, %EWL was 68.5 ± 19.9%, percent of total body weight loss (TWL%) was 30.1 ± 7.4%, and the BMI had decreased by 13.8 ± 3.7 kg/m^2^ (*p* < 0.001). Seventy-one (82.6%) participants took multivitamins every week, 81 (91%) were at a dietitian follow-up and 41 (46.6%) were under mental follow-up by a social worker o psychologist. Fifteen participants (29.4%, *n* = 51 respondents) reported having improved a personal hygiene problems after surgery. In addition, physical factors (snoring, pain affected by weight, and physical activity) had improved significantly (*p* ≤ 0.001). However, 10 (22.2%) participants, who did not suffer from pain before surgery, reported onset of chronic pain by the follow-uptime. The energy level increased by 24% from 61 ± 29.1 to 80.1 ± 21.2 post-surgery (*p* < 0.001), while the mood level had increased by 14% from 80.5 ± 21.5 to 91.5 ± 17.7 (*p* < 0.001). Social parameters also improved, with a decrease in social rejection from 13 (17.1%) pre-surgery to 4 (5.4%) post-surgery (*p* = 0.02), and an increase in participation in after-school social activities. The mental health score increased by 9.5% from 88.8 ± 21.8 pre-surgery to 97.2 ± 8.1 post-surgery (*p* = 0.002), although not all the individual components showed a significant improvement when calculated separately (Table 2).

**Table 2:** Changes in body weight and physical, social and mental parameters 9 months after metabolic bariatric surgery in adolescents.

	**Pre-surgery**	**Post-surgery**	**improvement in those with**	***N***	**P**
			**pre-condition**		
**Weight parameters**					
Weight (kg)	127.7 ± 17.9	89.1 ± 15.1	–	94	<0.001
BMI (kg/m^2^)	45.9 ± 5.9	32.2 ± 5.6	–	94	<0.001
**Physical and social parameters**					
Snoring*, *n* (%)	43 (56.6%)	6 (7.9%)	38 (88.3%)	76	<0.001
Pain*, *n* (%)	39 (46.4%)	16 (19.0%)	33 (84.6%)	84	0.001
Physical activity*, *n* (%)	35 (42.2%)	72 (86.7%)	40 (83.3%)^¥^	83	<0.001
After-school social activities*, *n* (%)	35 (47.3%)	52 (70.3%)	27 (69.2%)	74	0.008
Social rejection*, *n* (%)	13 (17.1%)	4 (5.4%)	11 (84.6%)	76	0.02
Energy level** (0–100 scale)	61.0 ± 29.1	80.1 ± 21.2	–	84	<0.001
Mood level** (0–100 scale)	80.5 ± 21.5	91.5 ± 17.7	–	82	<0.001
Mental health score***(0–**100 scale)**	88.8 ± 21.8	97.2 ± 8.1	–	50	0.002
Sadness^*^, n (%)	11 (13.9%)	3 (3.8%)	10 (90.9%)	79	0.039
Low self-esteem^*^, n (%)	8 (15.4%)	4 (7.7%)	6 (75%)	52	0.289
Hopelessness^*^, n (%)	14 (17.9%)	5 (6.4%)	13 (92.8%)	78	0.049
Suicidal thoughts*, n (%)	3 (5.6%)	0	3 (100%)	54	–
Self-hurt attempts*, n (%)	2 (3.6%)	1 (1.8%)	1 (50%)	55	1
*Smoking*	11 (12.9%)	19 (22.3%)	10 (13.3)^¥¥^	85	0.039

*Data in the table are presented as mean ± SD for numerical variables n (%) for dichotomous variables. Paired t- test was used to evaluate differences for numerical variables and McNemar test for nominal variables. All statistical analyses were two sided with a significance of <0.05. Pre-τsurgery weight and height were measured at the medical center on hospitalization. Post-surgery weight is based on self-reported data, post-surgery height was taken from the measurement of the medical center pre-surgery*.

*Dichotomous questions (yes/no). **Ordinal scale question (0–100 scale, higher score means better status). ***Sum of all the dichotomous questions below (0–100 scale, higher score means better mental health). ^¥^From those who did not performed any physical activity prior to surgery; ^¥¥^from those who did not smoke prior to surgery.

*BMI, body mass index; kg, kilograms; m, meters*.

Among the participants that underwent an additional surgery (*n* = 5), their EWL% was 73.5 ± 14.7% after a mean follow-up of 11 months (vs. 68.5 ± 19.9% after a mean of 9 months in the study group), and they visited 4.8 ± 2.2 times the ER, and were 3.3 ± 1/5 times hospitalized. Similar improvements in mental, physical, and social factors were found between those who underwent additional bariatric surgery and those with one metabolic bariatric surgery.

When comparing between sexes, males had a higher %EWL than females (75.7 ± 18.2% vs. 64.3 ± 19.7% respectively, *p* = 0.006). Social rejection improved significantly in females (*n* = 10, 83% of those presenting social rejection at baseline, *p* = 0.012), but not in males. Both females and males improved physical symptoms comparing to baseline, such as snoring (*n* = 23, 88%, *p* < 0.001 in those with snoring prior to the surgery for females and *n* = 15, 88%, *p* = 0.001 for males) and weight-related pain (*n* = 20, 83.3%, *p* = 0.019 for females and *n* = 13, 86.6%, *p* = 0.21 for males). Other parameters were comparable between males and females.

### Association Between %EWL and Baseline Parameters

We divided our sample into two categories, based on median %EWL: %EWL <67 (*n* = 48, mean of 53%), and %EWL ≥ 67 (*n* = 46, mean of 82%; Table 3). Three participants were excluded from the analysis (did not report weight after surgery). As expected, a long time from surgery to interview was associated with a higher %EWL (*p* = 0.010). More females achieved a lower %EWL than males (79.1% in the lower EWL% group compared to 45.6% in the higher EWL% group, *p* = 0.001), and those with a higher baseline BMI achieved a lower EWL compared to those with lower baseline BMI (*p* < 0.001). In addition, there were no associations detected between %EWL and changes in mental, physical, and social parameters.

**Table 3:** Baseline characteristics and quality of life changes post-surgery across percent excess weight loss categories.

	**EWL <67 %**	**EWL≥67 %**	***p***
	**n = 48**	**n = 46**	
**Baseline characteristics**			
Female gender	38 (79.1%)	21 (45.6%)	0.001
Baseline BMI, kg/m^2^	48.2 ± 6.4	43.6 ± 4.2	<0.001
Age, years	16.8 ± 1.0	17.3 ± 0.6	0.002
Time from surgery to interview, months	8.0 ± 3.0	9.8 ± 3.7	0.010
Physical, social and mental health changes			
Snoring (remission*)	19 (90.4%)	19 (86.3%)	–
	*n* = 21	*n* = 22	
Pain (remission*)	16 (84.2%)	17 (85.0%)	–
	*n* = 19	*n* = 20	
Physical activity (improvement**)	18 (81.8%)	22 (88.0%)	–
	*n* = 22	*n* = 25	
After-school social activities	12 (66.6%)	14 (70.0%)	–
(improvement**)	*n* = 18	*n* = 20	
Social rejection (remission*)	7 (77.9%)	4 (100%)	–
	*n* = 9	*n* = 4	
Δ Energy (0–100 scale)	19.5 ± 31.3	19.0 ± 43.4	0.956
	*n* = 41	*n* = 42	
Δ Mood level (0–100 scale)	10.0 ± 23.8	10.9 ± 25.0	0.858
	*n* = 40	*n* = 41	
Δ Mental health score	8.4 ± 18.0	8.3 ± 18.5	0.980
(0–100 scale)	*n* = 26	*n* = 24	

*Group division is based on EWL% median = 67%. Data in the table are presented as mean ± SD for numerical variables and n (%) for dichotomous variables. T-test was used to evaluate differences for numerical variables and Chi square test for nominal variables. P is missing if one or more cells have an expected count <5. All statistical analyses were two sided with significance of <0.05. %EWL was calculated from measured height and weight at baseline, and from the post-surgery self-reported weight. Total N is 94, as three participants do not have post-surgery weight self-report. N is listed for a parameter when not all the group was relevant / data was missing. Energy level, mood level and mental health score are 0–100 scales; higher score means a better status*.

*From those who answered “yes” at baseline. **from those who answered “no” at baseline.

*%EWL, Percent excess weight loss; BMI, Body mass index; Δ, (post-surgery score) – (pre-surgery score)*.

### Association Between Changes in Mental Health Parameters and Baseline Parameters

Associations between changes in energy and mood levels, mental health score, physical and social factors, and independent variables, such as age, gender, ethnic group, and baseline BMI were examined by a univariate linear regression model.

The change in mental health score was significantly associated to baseline social rejection (r = 0.514, *p* < 0.001), meaning that the mental health score of participants, who reported social rejection at baseline, improved more than those who did not report experiencing this condition. This association remained significant after adjusting for age, gender, and baseline BMI. The results were similar after the sensitivity analysis and baseline weights values (reported vs. measured) were highly correlated (*r* = 0.82, *p* < 0.001).

## Discussion

In this short-term nationwide prospective cohort study, we found that MBS in adolescents led to a 30% reduction in BMI and an increase in physical, mental, and social parameters. However, the improvement in those QoL factors was not associated with %EWL.

All physical parameters in our study, including snoring, pain related to weight, physical activity, and energy level, improved postoperatively following MBS, by previous literature's physical subscales (19, 21–23). A study that evaluated the quality of life using SF-36 on adolescents showed that reflux, nausea, and diarrhea after surgery worsened physical status over time, but remained higher than baseline and showed that the highest improvement after surgery was related to physical functioning, physical role functioning and general health (19). Mental health has shown to be similar in adolescents after MBS compared to adolescents with obesity taking part in a non-surgical lifestyle modification program (23). Up to 5 years after surgery, mental health status has remained similar (19) and has improved mainly concerning mental functioning scores (22) or partially concerning two of four mental health domains (*n* = 81) (8). Similarly, our findings showed an improvement in mental health scores, mainly in 2 domains, in addition to all physical and social factors. Similar to previous studies, the rate of weight loss was not associated with social, physical, and mental parameter changes in our study. A previous review of 10 studies (*n* = 322) (24) and studies evaluating adolescents' mental health (25) and psychopathology (26) 2 years following surgery showed that changes in BMI did not necessarily correlate with improvement in quality of life. However, another study reported that a greater percentage of weight loss was associated with better weight and physical quality of life 2 years post-surgery (23). Another large study of 242 adolescents reported 6% lower odds of having musculoskeletal pain and 10% lower odds of having lower extremity pain per 10% reduction of BMI postoperatively (21). In our study, the significance of this association was difficult to determine (including snoring, pain, and physical activity) probably due to the sample size and change in pain is mainly relevant for those who reported the condition at baseline. Since most physical functioning questions in our study are dichotomous and considering previous literature findings, the association between %EWL and physical patterns, especially pain, might exist. Additionally, our study showed an increase in smoking percentage in accordance with an American cohort that showed a rise in smoking habits in teens who underwent bariatric surgery and those who did not (27).

We detected a meaningful correlation between improvement in mental health scores and baseline social rejection. This finding emphasizes the importance of peer relationships in youth (28) and their meaningful contribution to mental health improvement among teenagers undergoing MBS. It also reflects the potential impact of obesity stigma on teens seeking MBS and the opportunity for health status improvement for those who experience stigmatization. The impact of weight-related stigma was mentioned elsewhere (29, 30) and was unrelated to BMI and associated with poorer weight-related quality of life.

There are several limitations in the design of this study. The questionnaire was written for parents and for adolescents, who mainly answer it together and there is no record of whether the responder is a parent or an adolescent. The pre-surgery questionnaire was self-completed by the adolescents with their parents without the mediation of an interviewer, and the post-surgery questionnaire was completed by phone interview. Despite that the questionnaire was not fully validated, the sensitivity analysis for the weight report suggested that the reported weights were reliable. In addition, the information regarding the reason for the additional bariatric surgery of five participants was missing. Although these limitations are meaningful, the main results on physical and mental factors are consistent with literature reports, assuming that the questions add valuable data. In addition, the time from surgery to interview varied with a range of 4–16 months, while a higher %EWL was associated with a longer time from surgery to interview. This finding provides a logical directionality for the weight changes. This short-term represents mainly the weight loss phase, and as shown previously in the AMOS study, a longer follow-up of 2 years may show a slow-down in weight loss (8, 11) and weight regain in some patients. To reduce the effect of time from surgery was inserted into the multivariate regressions model to adjust this possible confounder. Another limitation of the study is the finding that more males refused to be interviewed than females, which could lead to a possible selection bias, although baseline characteristics were mainly similar, and the analyses were adjusted for gender. This higher proportion of females reflect the bariatric population. It is important to point out that these results reflect physical, social factors, and mental health of the ongoing weight loss phase. Further studies should examine this scope in a longer-term after the surgery.

## Conclusion

The MBS in adolescents, during the weight loss phase, leads to a meaningful decrease in BMI and improvement in physical, mental, and social factors. Although the correlation to EWL% was not found, patients experiencing social rejection may benefit from MBS, especially in short-term mental health. Further studies should investigate mental, physical, and social parameters in a longer-term post-surgery.

## Data Availability Statement

The data that support the findings of this study are available on request from the corresponding author (YG).

## Ethics Statement

The studies involving human participants were reviewed and approved by the Institutional Review Board of the Tel HaShomer Medical Center (SMC-6199-19). Written informed consent to participate in this study was provided by the participants' legal guardian/next of kin.

## Author Contributions

TM, RV-K, OZ, and YG conceived and carried out the study. TM, AG, YG, and OZ analyzed the data and wrote the manuscript. All authors approved the final version of the manuscript.

## Conflict of Interest

The authors declare that the research was conducted in the absence of any commercial or financial relationships that could be construed as a potential conflict of interest.

## Publisher's Note

All claims expressed in this article are solely those of the authors and do not necessarily represent those of their affiliated organizations, or those of the publisher, the editors and the reviewers. Any product that may be evaluated in this article, or claim that may be made by its manufacturer, is not guaranteed or endorsed by the publisher.
