# Supplementary material for: Short-Term Changes in Mental, Physical, and Social Factors After Metabolic Bariatric Surgery in Adolescents: A Nationwide Prospective Cohort Study
Source: Front Nutr. 2022 May 12;9:878202. doi: 10.3389/fnut.2022.878202 (PMC9133935; doi:10.3389/fnut.2022.878202)
Supplement: Supplementary file 1 [file Table_1.docx]

**Table S1. Baseline characteristics of adolescents who were interviewed compared to those who refused to be interviewed**

| **P** | **Refused (n=16)** | **Interviewed (n=97)** |  | |
| --- | --- | --- | --- | --- |
|  |  |  | **Demographic characteristics** | |
| 0.001 | 3 (18.8%) | 62 (63.9%) | Female gender, n (%) | |
| 0.65 | 17.1±0.6 | 17.0±0.9 | Age, years | |
| 0.69^*^ | 15 (93.8%) | 82 (84.5%) | Jewish | Ethnic group,  n (%) |
|  | 1 (6.3%) | 14 (14.4%) | Arabic |  |
|  | *n=16* | *n=96* |  |  |
|  |  |  | **Clinical characteristics** | |
| 0.131^**^ | 11 (68.8%) | 84 (86.6%) | Sleeve gastrectomy | Surgery procedure, n (%) |
|  | 3 (18.8%) | 10 (10.3%) | Omega loop, mini |  |
|  | 0 | 2 (2.1%) | Roux-en Y gastric bypass |  |
|  | 2 (12.5%) | 1 (1%) | Gastric band |  |
| 0.56 | 47.0±6.9 | 46.1±5.9 | Baseline BMI, kg/m^2^ | |
|  |  |  | **Comorbidities** | |
| 0.39^*^ | 3 (18.8%) | 10 (10.3%) | Diabetes, n (%) | |
| 0.462^*^ | 1 (6.3%) | 15 (15.5%) | Hypertension, n (%) | |
| 0.23^*^ | 2 (12.5%) | 29 (29.9%) | Dyslipidemia, n (%) | |
| 0.56 | 9 (56.3%) | 65 (67.0%) | Fatty liver, n (%) | |
|  | *n=15* | *n=96* |  |  |
| 1^*^ | 4 (25%) | 25 (25.8%) | Obstructive sleep apnea, n (%) | |
| - | 0 | 4 (4.1%) | Anxiety, n (%) | |
|  | *n=14* | *n=93* |  |  |
| 0.57^*^ | 1 (6.3%) | 5 (5.2%) | Depression, n (%) | |
|  | *n=14* | *n=94* |  |  |
| 0.43^*^ | 3 (18.8%) | 11 (11.3%) | Smoking, n (%) | |
|  | *n=97* | *n=94* |  |  |
| 0.001 | 141±17 | 127±15 | SBP, mmHg | |
|  | *n=97* | *n=96* |  |  |
| 0.23 | 77.8±16 | 72.8±11 | DBP, mmHg | |
|  | *n=97* | *n=96* |  |  |
|  |  |  | **QoL, baseline parameters** | |
| 0.84 | 8 (50%) | 50 (51.5%) | Snoring, n (%) | |
|  | *n=15* | *n=89* |  |  |
| 0.69^*^ | 1 (7.1%) | 14 (17.1%) | Social rejection, n (%) | |
|  | *n=14* | *n=82* |  |  |
| 0.84 | 65.0±29.6 | 63.3±29.0 | Energy level (0-100 scale) | |
|  | *n=97* | *n=94* |  |  |
| 0.99 | 81.2±21.4 | 81.3±21.3 | Mood level (0-100 scale) | |
|  | *n=97* | *n=91* |  |  |
| 0.77 | 85.7±19.9 | 87.5±23.2 | Mental health score (0-100 scale) | |
|  | *n=14* | *n=75* |  |  |
|  |  |  | Weight loss characteristics | |
| 0.594 | 62.7±26.6 | 57.9±17.2 | EWL% 6 months after surgery | |
|  | *n=10* | *n=60* |  | |

Data in the table are presented as mean ± SD for numerical variables and n (%) for dichotomous variables. *t*-test was used to evaluate differences for numerical variables and Chi – square test for nominal variables. All statistical analyses were two sided with a significance of <0.05. N is listed when some of the data are missing. Energy level, Mood level and Mental health score are 0-100 scales, higher score means a better QoL. ^*^ calculated by Fisher's exact test. ^**^ calculated as sleeve gastrectomy vs. others, by Fisher's exact test. Abbreviations: SBP, systolic blood pressure; DPB, diastolic blood pressure; kg, kilograms; m, meters; mmHg, millimeters of mercury.
