# Supplementary material for: Short-Term Changes in Mental, Physical, and Social Factors After Metabolic Bariatric Surgery in Adolescents: A Nationwide Prospective Cohort Study
Source: Front Nutr. 2022 May 12;9:878202. doi: 10.3389/fnut.2022.878202 (PMC9133935; doi:10.3389/fnut.2022.878202)
Supplement: Supplementary file 3 [file Data_Sheet_1.DOCX]

**Supplementary 1. Questionnaire of mental, physical and social factors before and after bariatric surgery**

1. Surgery date _________
2. Body Height (cm) _________
3. Body Weight (Kg) _________
4. Weight on surgery day (Kg) _______
5. Did you visited the dietitian since the surgery? Yes / no (if your response is no, please continue to question #9)
6. Since the surgery, how many times did you visited the dietitian? _____
7. Where did you visited the dietitian (you can choose more than one answer)
   1. In the hospital were the surgery was performed
   2. At the community medicine
   3. Private
   4. Other: __________
8. Did you receive funding by the Health Maintenance Organization for the dietitian appointments?
9. Why you did not visited a dietitian?
   1. Lack of funding by the Health Maintenance Organization
   2. Distance to the dietitian Clinic
   3. Time limitation
   4. Lack of awareness of the need of follow up / We were not referred to the dietitian
   5. Not feel that it is necessary, I do well without the follow-up
   6. Other _______
10. Please answer if you had any of the following symptoms since the surgery
    1. Snoring: yes / no
    2. Pain related to body weight: yes / no
    3. General energy level: very high 5 4 3 2 1 very low
    4. Mood in general: Very good 5 4 3 2 1 Very deteriorated
    5. Goes to school regularly: yes / no
    6. Involved in social activities at school: yes / no
    7. Participate in after-school activities: yes / no
    8. Victim of bulling or social rejection: yes / no
    9. Feels sad most of the days: yes / no
    10. Feel hopeless moments: yes / no
    11. Participate in physical activities: yes / no
    12. Suffer from low self-esteem: yes / no
    13. Has self-injury thoughts: yes / no
    14. Has experienced a self-injury attempt: yes / no
    15. Experiences self-hygiene problems: yes / no
11. Please fill in the table your supplement habits:

| Supplement type | Do not take | Daily intake | 1-3 times a week | 1-3 times a month |
| --- | --- | --- | --- | --- |
| Calcium |  |  |  |  |
| Calcium + vitamin D |  |  |  |  |
| Vitamin D |  |  |  |  |
| B12 |  |  |  |  |
| B1 |  |  |  |  |
| Iron |  |  |  |  |
| Folic Acid |  |  |  |  |
| Multivitamin |  |  |  |  |

1. Medical information

| Has your child been diagnosed with one of the following | Before surgery | Medication intake | After surgery | Medication intake |
| --- | --- | --- | --- | --- |
| Hypertension | yes / no | yes / no | No change  Improvement  worsen | yes / no |
| Diabetes | yes / no | no / diet / pills / insulin | No change  Improvement  worsen | no / diet / pills / insulin |
| Hyperlipidemia | yes / no | no / diet / pills | No change  Improvement  worsen | no / diet / pills |
| Obstructive Sleep Apnea | yes / no | no / pills / C-PAP | No change  Improvement  worsen | no / pills / C-PAP |
| Irregular periods (for women) | yes / no | yes / no | No change  Improvement  worsen | yes / no |
| Fatty liver | yes / no | yes / no | No change  Improvement | yes / no |
| Pseudotumor-cerebri | yes / no | yes / no | No change  Improvement | yes / no |
| Anxiety | yes / no | yes / no | No change  Improvement | yes / no |
| Depression | yes / no | yes / no | No change  Improvement | yes / no |
| Attention-Deficit Hyperactivity Disorder (ADHD) | yes / no | yes / no | No change  Improvement | yes / no |
| Eating habits (overeating episodes, induced vomiting, bulimia, binge eating disorder) | yes / no | yes / no | No change  Improvement | yes / no |

1. Have you been under mental follow-up by a social worker o psychologist since surgery?
2. Where have you visited a mental health professional? _________
3. How many visits did you have since surgery? ___
4. Have you participated in support groups since surgery? Yes / No.
5. How many group meetings have you assisted? _______

**Complications after surgery**

1. Have you visited the E.R since surgery? Yes (continue to question #19) / No (continue to question # 20).
2. How many times have you visited the E.R since surgery? ____
3. Have you been hospitalized since surgery? Yes (continue to question # 21) / No (continue to question #22).
4. How many times have you been hospitalized since surgery? ____
5. Did you underwent additional surgical procedures since the bariatric surgery? Yes / No.
6. Do you smoke? Yes (continue to the following question) / No (continue to question #26).
7. How many cigarettes per day? ___
8. For how long (years) you smoke? ___
9. Have you ever smoked before? Yes / No.
10. Do you regularly consume alcoholic beverages? Yes / No.
